# Fully Characterized Effective Bacteriophages Specific against Antibiotic-Resistant *Enterococcus faecalis*, the Causative Agent of Dental Abscess

**DOI:** 10.3390/medicina60030501

**Published:** 2024-03-19

**Authors:** Asmaa Ramadan, Mohamed O. Abdel-Monem, Noha K. El-Dougdoug, Alsayed E. Mekky, Shymaa A. Elaskary, Abdulaziz A. Al-Askar, Shimaa A Metwally, Ahmed F. El-Sayed, Gehad AbdElgayed, Ebrahim Saied, Mohamed Khedr

**Affiliations:** 1 Botany and Microbiology Department, Faculty of Science, Benha University, Benha 13511, Egypt; asmaarm@yahoo.com (A.R.); momonem2003@yahoo.com (M.O.A.-M.); nohaeldougdoug@gmail.com (N.K.E.-D.); 2 Botany and Microbiology Department, Faculty of Science, Al-Azhar University, Nasr, Cairo 11884, Egypt; alsayedessam@azhar.edu.eg (A.E.M.); hema_almassry2000@azhar.edu.eg (E.S.); 3 Medical Microbiology and Immunology Department, Faculty of Medicine, Menoufia University, Shibin El Kom 32511, Egypt; 4 Botany and Microbiology Department, Faculty of Science, King Saud University, P.O. Box 2455, Riyadh 11451, Saudi Arabia; aalaskara@ksu.edu.sa; 5 Microbiology and Immunology Department, Faculty of Pharmacy for Girls, Al-Azhar University, Cairo 4434004, Egypt; dr.shimaaabdelrahman@azhar.edu.eg; 6 Microbial Genetics Department, Biotechnology Research Institute, National Research Centre, Giza 12622, Egypt; ahmedfikry.nrc@gmail.com; 7 Egypt Center for Research and Regenerative Medicine (ECRRM), Cairo 11517, Egypt; 8 Integrated Molecular Plant Physiology Research, Department of Biology, University of Antwerp, 2020 Antwerp, Belgium; gehad.hegazygadabdelgayed@uantwerpen.be

**Keywords:** bacterial dental abscesses, *Enterococcus faecalis*, *E. faecalis*_phage-01, *E. faecalis*_phage-02, multi-drug resistance, oral epithelial cell line

## Abstract

*Background and Objectives*: *Enterococcus faecalis (E. faecalis)* is a primary pathogen responsible for dental abscesses, which cause inflammation and pain when trapped between the crown and soft tissues of an erupted tooth. Therefore, this study aims to use specific phages as an alternative method instead of classical treatments based on antibiotics to destroy multidrug-resistant *E. faecalis* bacteria for treating dental issues. *Materials and Methods*: In the current study, twenty-five bacterial isolates were obtained from infected dental specimens; only five had the ability to grow on bile esculin agar, and among these five, only two were described to be extensive multidrug-resistant isolates. *Results*: Two bacterial isolates, *Enterococcus faecalis* A.R.A.01 [ON797462.1] and *Enterococcus faecalis* A.R.A.02, were identified biochemically and through 16S rDNA, which were used as hosts for isolating specific phages. Two isolated phages were characterized through TEM imaging, which indicated that *E. faecalis*_phage-01 had a long and flexible tail, belonging to the family *Siphoviridae*, while *E*. *faecalis*_phage-02 had a contractile tail, belonging to the family *Myoviridae.* Genetically, two phages were identified through the PCR amplification and sequencing of the RNA ligase of *Enterococcus* phage vB_EfaS_HEf13, through which our phages shared 97.2% similarity with *Enterococcus* phage vB-EfaS-HEf13 based on BLAST analysis. Furthermore, through in silico analysis and annotations of the two phages’ genomes, it was determined that a total of 69 open reading frames (ORFs) were found to be involved in various functions related to integration excision, replication recombination, repair, stability, and defense. In phage optimization, the two isolated phages exhibited a high specific host range with *Enterococcus faecalis* among six different bacterial hosts, where *E. faecalis*_phage-01 had a latent period of 30 min with 115.76 PFU/mL, while *E*. *faecalis*_phage-02 had a latent period of 25 min with 80.6 PFU/mL. They were also characterized with stability at wide ranges of pH (4–11) and temperature (10–60 °C), with a low cytotoxic effect on the oral epithelial cell line at different concentrations (1000–31.25 PFU/mL). *Conclusions*: The findings highlight the promise of phage therapy in dental medicine, offering a novel approach to combating antibiotic resistance and enhancing patient outcomes. Further research and clinical trials will be essential to fully understand the therapeutic potential and safety profile of these bacteriophages in human populations.

## 1. Introduction

Tooth abscesses or periapical infections usually occur because of tooth decay, trauma, or failed treatment of the root canal [1]. Infections left untreated not only cause excruciating pain, but also carry the significant danger of spreading to the throat or the brain. Abscessed tooth identification and treatment not only alleviate symptoms, but also prevent dangerous repercussions [1,2]. By destroying the tooth’s protective enamel, oropharyngeal bacteria can enter the tooth’s (pulp) cavity and cause local infection [3,4]. As inflammation accompanies the infection that grows in the pulp cavity within the confined cavity of the tooth, it presses against the lining of the dentin, causing severe pain. Another factor that predisposes to tooth abscess is a partially injured tooth, most frequently a wisdom tooth, where bacteria are trapped between the soft tissue and the crown, causing inflammation [5,6]. Treatment includes draining the abscess, administering antibiotics, controlling pain, and removing the infection source within the tooth. Oral antibiotics with a timely consultation with a dentist are often sufficient for dental treatment in patients [7]. *E. faecalis* is one example of a pathogen that is hard to eradicate in dentistry and is one of the most known causes of recurrent failures of root canal treatment [8]. Its positive reactant is Gram’s stain, as a non-spore-forming bacterium that normally occurs in the human oral cavity [9]. Different antiseptics, aside from antibiotics, are used for bacterial elimination inside the canal, either through calcium hydroxide or antibiotic pastes, to enhance bacterial control and seal the root canal, but *E. faecalis* is known for its high resistance to various antimicrobial agents, which develop through both innate and acquired mechanisms. It is resistant to almost all cephalosporins, clindamycin, and trimethoprim–sulfamethoxazole aminoglycosides [10,11]. Owing to evolving resistance, most over-the-counter antibiotics are ineffective against *E. faecalis* infections, so it is urgent for global health that alternative antibacterial agents be evaluated.

Phage treatment is more effective than antibiotics in eliminating MDR microorganisms for several reasons [12], including the remarkable specificity of phages to the host without disrupting the normal microbiota [13]. Additionally, phages tend to replicate until the host bacteria’s population density is reduced and remain infectious under very harsh environmental conditions [14]. Furthermore, phages are not toxic to mammals or humans and can be used for patients with antibiotic allergies [15]. Additionally, compared with the cost of producing novel antibiotics, generating phage preparations is inexpensive, it is easy to isolate new phages from a wide range of sources [16,17], and phages have the efficacy to penetrate and destroy bacterial biofilms [18]. Phage therapy has been evaluated with multi-drug resistant oral bacteria during animal and human studies and has been found to be safe [19].

Our study aims to isolate, purify, propagate, and characterize the lytic *E. faecalis* phage to eliminate dental abscess-causative multi-drug resistant *E. faecalis.*

## 2. Materials and Methods

### 2.1. Chemicals Used

The chemicals and reagents, chloroform, elution buffer, wash buffer, agarose gel, SM buffer, NaOH, HCl, NaCl, polyethylene glycol 8000 (PEG 8000), λ buffer, DMSO, brain heart infusion broth medium, bile esculin agar, nutrient agar, MuellerHinton agar, Luria Bertani (LB) broth, and TSA medium were of AR grade and procured from Sigma-Aldrich, Cairo, Egypt. All biological syntheses in the current study were achieved using distilled water (dis. H_2_O).

### 2.2. Isolation of Enterococci

Twenty-five sterile cotton swabs were collected from patients (15 females and 10 males) ranging in age from 20 to 45 years over a period of 7 months from five outpatient clinics of Menoufia Hospitals. The swabs were distributed as 14 from abscesses, 8 from caries, and 3 from gingivitis. The clinical specimens were immediately transported to the laboratory of the Medical Microbiology and Immunology Department, Faculty of Medicine, Menoufia University, using brain heart infusion broth medium for bacteriological analysis [20]. All samples were streaked on bile esculin agar as a selective medium for *Enterococcus* isolates and on blood agar plates to identify their blood hemolysis type [21].

### 2.3. Ethical Approval and Consent Form

After 24 h of incubation, 25 bacterial isolates were grown on nutrient agar plates; according to the Gram reaction, they were divided into 15 Gram-positive and 10 Gram-negative samples for selecting *Enterococci*, and a specific and selective medium bile esculin agar was chosen to cultivate fifteen Gram-positive isolates. Out of the fifteen Gram-positive isolates, ten with colonial characteristics of *Enterococci* were inoculated in slants and stored at 4 °C for further work. The study was approved by the Ethical Committee of Human Rights of Research at Menoufia University (IRB approval number and date 12/2023MICR4-1), in accordance with the Declaration of Helsinki. A written informed consent form was signed by each participant included in this study after informing them about the study. All data have been kept and preserved.

### 2.4. Identification of Enterococcus spp.

The Gram-positive samples were sub-cultured three times in succession on bile esculin medium for the selection and purification of *Enterococci*. *Enterococcus* colonial morphology, Gram staining, and other traditional biochemical assays were used to identify isolates. (Clinical isolates were also detected utilizing the VITEK^®^2 system BioMérieux (Version 8.01).

### 2.5. Antibiotic Susceptibility Test

The Kirby–Bauer technique was used to test the antibiotic susceptibility of Gram-positive *Enterococci* grown on bile esculin [22]. Ten antibiotic discs were used in this assay as follows: aztreonam (30 μg), nalidixic acid (30 μg), bacitracin (0.04 μg), clindamycin (2 μg), imipenem (10 μg), fusidic acid (10 μg), norfloxacin (10 μg), streptomycin (10 μg), B. ofloxacin (5 μg), and vancomycin (30 μg). After inoculating the bacteria via a sterile swab onto Mueller–Hinton agar, the antibiotic disc was then placed on the inoculated agar plate with forceps. The plate was turned upside down and left in a 37 °C incubator for 18–24 h. The findings were validated using the Clinical and Laboratory Standards Institute’s recommendations [23].

### 2.6. Bacterial Genomic Identification through 16S rRNA Sequencing

The most extensive antibiotic-resistant *Enterococci* were cultivated overnight in Luria Bertani (LB) broth according to the methodology by Alsamman et al. [24] for genomic DNA extraction. The following universal bacterial primers were used: 5′-AGA GTT TGA TCC TGG CTC AG-3′ alongside another reverse primer 5′-GGT TAC CTT GTT ACG ACT T-3 [25], synthesized by Sigma Scientific Services Co., 6 October City, Egypt, targeting 16S rRNA gene amplification via PCR. The PCR reaction mixture was composed of 25 µL Master Mix, which was Hot Start PCR Master Mix, 2X, with 20 µmol of each primer in a reaction volume of 50 µL. The PCR reaction was carried out under the following conditions: the first cycle denaturation step was at 95 °C for 10 min, followed by 35 cycles of 95 °C for 30 s, 65 °C for 1 min, 72 °C for 1 min, and finally the last cycle with extension at 72 °C for 10 min.

#### Purification and Analysis of the PCR Product

Amplicons of DNA were purified via the GeneJET PCR Purification Kit (Thermo, CA, USA), as follows: 45 µL of binding buffer was thoroughly mixed with the PCR product mixture; the mixture was transferred to a GeneJET™ Purification Column, centrifuged at 14,000× *g* for 1 min, and the flow-through was discarded. One-hundred microliters of wash buffer were added to the GeneJET ^TM^ Purification Column, then centrifuged for 1 min, and the flow-through was discarded. Finally, the purified PCR products were eluted using 25 µL of elution buffer, centrifuged at 14,000 rpm for 1 min, and the eluted DNA was stored at −20 °C until use. Purified DNA products were sequenced using capillary electrophoresis with an ABI 3730xl DNA sequencer (GATC, Irvine, CA, USA). Raw sequence data analysis was performed using sequencing analysis software.

### 2.7. Bacteriophage Isolation

Isolation and propagation of *E. faecalis* phages were performed through cultivation on two *Enterococci* clinical isolates selected as extensive MDR, *E. faecalis* A.R.A.01 and *E. faecalis* A.R.A.02, as specific hosts. The enrichment process was used to isolate *E. faecalis* phages from several sewage water samples collected in Giza, Egypt. Chloroform was used to treat the sewage samples; then, they were centrifuged at 10,000 rpm for 15 min. After particle removal using filter paper, 50 mL of the filtrate was added to 100 mL of TSB medium, and 1 mL of overnight culture of *E. faecalis* was cultured for 24 h at 37 °C in a shaking incubator at 120 rpm. The culture was treated with chloroform, then centrifuged at 10,000 rpm for 15 min to isolate the possible phages. Bacterial lawns of *E. faecalis* were added to the surface of TSA plates through the double agar overlay technique, as described by Thung et al. [26] with some modifications. In brief, 100 µL of bacterial culture was mixed with 5 mL of semi-solid TSB and poured over solid TSA agar plates. After drying, 15 µL of a pre-prepared putative phage source was spotted on the lawns. The plates were incubated at 37 °C for 24 h, then examined for plaque formation.

#### 2.7.1. *Purification of Phages*

According to Thung et al. [26], phages were isolated and cultured subsequently from plaque isolates, and these plaques were inoculated in 1 mL of nutrient broth, which included 100 µL of two host bacterial isolates, and then incubated at 37 °C with a speed of 120 rpm on an incubator shaker. After incubation, chloroform was added, and the mixture was centrifuged again at 10,000 rpm for 15 min. The supernatants containing our phages were purified via filtration through a 0.22 μm Millipore filter membrane (Minisart, Sartorius, Songdo, Republic of Korea) and then stored as bacteriophage stock at a low temperature (4 °C). Then, 0.5 mL of supernatant was mixed with 1 mL of bacteria and incubated at 37 °C for 30 min. Then, the mixture was added to 3 mL of semi-solid TSB and poured over solid TSA agar plates. The plates were kept at 37 °C for one day, then examined for lysis zone formation.

#### 2.7.2. Characterization of *E. faecalis* Phage

##### Transmission Electron Microscopy (TEM)

Morphological characterization of isolated phages was performed via a Hitachi transmission electron microscope H-9500 (Tokyo, Japan) at the Faculty of Science, Al-Azhar University, Cairo, Egypt. The phage samples were prepared and examined by TEM [27].

#### 2.7.3. *Genomic Identification of Phages*

##### PCR Conditions

The total genomic viral DNA was isolated from the selected phages using the Wizard Genomic DNA Kit (Promega, Madison, WI, USA). The methodology was in accordance with the instructions of the manufacturer. Four primers were designed through NCBI primer BLAST based on the gene (vBEfaSHEf13_001) of *Enterococcus* phage vB_EfaS_HEf13 with accession number (AYH92657.1), as shown in Table 1. The PCR conditions were: 32 cycles, including a denaturation stage at 94 °C/30 s, annealing at 58.3 °C/60 s, extension at 72 °C/90 s, and a final extension at 72 °C/180 s.

DNA amplicons were visualized using 1% agarose gel, 10 µL aliquots were electrophoresed, and the correct molecular weight of PCR amplicons (about 1800 base pair fragments) was confirmed. Gels were analyzed and captured on camera using UV [28]. QI Quick spin columns were used to purify the PCR products (Qiagen Inc., Chatsworth, CA, USA). According to a previous study [28], purified PCR amplicons were examined utilizing a Perkin Elmer 377 DNA sequencer, and the Dye Deoxy Terminator Cycle Sequencing Kit (Perkin Elmer, Foster City, CA, USA) was used for sequencing. The BLAST tool of GenBank was used to identify phage species with comparable gene sequences. A phylogenetic tree was constructed for each phage BLAST result against the top 10 similar sequences identified in the NCBI database and the relative similarities among the isolated virus and other viruses identified in the BLAST result were determined [29].

##### Bioinformatics and In Silico Analysis of *Enterococcus* Phages

The predicted genes were subjected to annotation using RAST v2 and were further searched in the UniProt database. The BLASTX algorithm was employed to filter out hits based on the E-value, identity, and score of each gene. This process utilized the online Uniprot database with default settings for comparison and identification. To assess the presence of virulence factors, antibiotic resistance genes, CRISPR, and CRISPR-like systems in the phage genome, the sequence was analyzed using the Virulence Factor Database (VFDB), the Comprehensive Antibiotic Resistance Database (CAR), and CRISPR-Cas Finder, respectively [30]. For phylogenetic analysis, a neighbor-joining phylogenetic tree was constructed using MEGA 11 software v 11.0.13, based on the sequence alignment of the phage of interest and the most closely related phages [31].

#### 2.7.4. *Phage Optimization*

##### One-Step Growth Curve

The latent period of phages and burst size were estimated as described by Kropinski et al. [32]. A known number of log-phase culture (1 × 10^7^ CFU/mL) of each bacterial host were separately added to each distinct phage (1 × 10^5^ PFU/mL) to achieve MOI = 0.01 and incubated at room temperature for 5 min for adsorption. The mixture of bacterial and phage suspensions was diluted (10^2^, 10^3^, and 10^4^ dilutions) and incubated at 37 °C. After 7 min, suspensions were diluted and then plated by mixing 100 μL of suspension with 4 mL of overlay medium, which included 10 ^8^ cfu/mL of bacterial isolates, and pouring the mixture onto TSA plates. The number of plaques was determined after 24 h of incubation at 37 °C. Relative burst sizes were determined using the following equation:

Relative burst size = final titer/start titer

The relative burst size at different times was plotted against time to determine the latent period and burst size.

##### Phage Host Range

We assessed phage activity against various bacterial species. Suitable media-containing double-layer agar plates were inoculated with individual doses of *Enterococcus faecium*, *Pseudomonas aeruginosa*, *Streptococcus mutans*, *Staphylococcus aureus*, and *E. coli.* As previously mentioned, on TSA plates, 10 µL of each phage suspension was spotted on the lawns. After 24 h at 37 °C, the plaques on the plates were analyzed [33].

##### Phage Thermal and pH Stability

Phage thermal stability was measured by adding 100 µL of phage lysate to prewarmed 0.22 m filter-sterilized SM buffer. For a period of one hour, the tubes were incubated at different temperatures ranging from 10 °C to 80 °C. Aliquots were obtained and phage titers were calculated after 60 min of incubation. To test pH stability, sterile SM buffer with pH values ranging from 2 to 13 that had been altered with NaOH and HCl was added to 100 µL of phage lysates. Sixty minutes were spent incubating the tubes at 37 °C. The two-layer agar method was used to detect the phage titers in bacterial hosts after the phage solution had been serially diluted [33]. The median of the triplicate counts was obtained for each temperature, besides the pH treatment that was carried out in triplicate. Phage heat/pH stability (%) = initial phage titer before treatment × 100% × (phage titer regained after treatment).

##### Production of Concentrated Purified Phage

Using the agar overlay technique [26], crude lysates were counted and viability was determined prior to use. Each crude lysate was treated with NaCl to obtain a final concentration of 1 M and stored at −20 °C for 1 h, followed by centrifugation at 11,000× *g* and 4 °C for 10 min. Following centrifugation, the supernatant was treated with 10% (*w*/*v*) polyethylene glycol 8000 (PEG 8000) and stored at 4 °C for 18 h. The suspensions were centrifuged at 11,000× *g* for 10 min at 4 °C, the supernatant was discarded, and the pellet was resuspended in 1 mL of chloroform and 11 mL of λ buffer. Centrifugation at 3000× *g* and 4 °C for 15 min separated the organic and aqueous phases. The organic phase was removed after centrifugation at 3000× *g*/15 min and 4 °C, and the aqueous phase was made up to 50 mL with buffer λ, then filtered twice through 0.45 μm filters. Purified phage preparations were diluted in λ buffer to achieve an active solution with a concentration of 8 × 10^9^ PFU/mL and quantified using the agar overlay method as previously described [26]. Buffer was stored in a sterile glass container at 2–8 °C. Before being used in cytotoxicity experiments, phages were diluted in buffer λ to yield 2 × 109.2 × 10^8^ and 2 × 10^7^ pfu/mL and then weighed by suspension at ordinary temperature.

##### Determination of Phage Cytotoxicity on Cells (MTT Assay)

Our procedure was followed as described previously by Mekky et al. [34]. The cell monolayer was washed twice with wash media and partial or complete loss of the monolayer, rounding, shrinkage, or cell granulation were determined. Twenty microliters of MTT solution were added to each well and placed on a shaking table (150 rpm for 5 min) to thoroughly mix the MTT into the media. The plate was incubated for 4 h at 37 °C under 5% CO_2_ to allow the MTT to be metabolized. In 200 µL of DMSO, Formazan (MTT metabolic product) was resuspended, and the plate was shaken for 5 min at 150 rpm to fully mix the formazan into the solvent. The optical density should be proportional to the number of cells.

### 2.8. Statistical Analysis

The data collected in the present study are presented as the means of three independent replicates and subjected to statistical analysis; standard deviation and standard error statistics were calculated via ANOVA, a one-way factor incorporated into Microsoft Excel 2016.

## 3. Results

### 3.1. Isolation of Enterococci

All 25 bacterial isolates were from clinical specimens, 10 males and 15 females, with an age range of twenty to forty-five years old. All patients were suffering from dental abscesses and had not received any antibiotics yet.

The isolation process was performed from three different sources, 14 isolates from abscesses, 8 from caries, and 3 from gingivitis.

**Figure 1:**
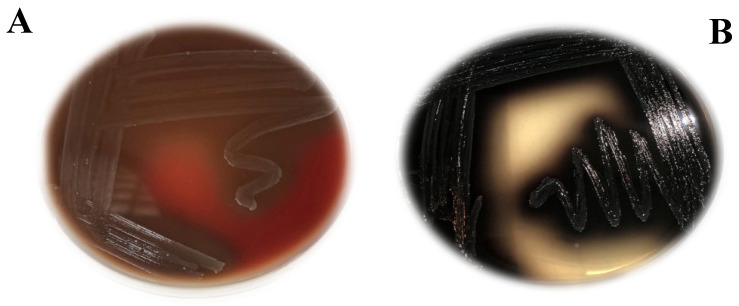
(**A**) *Enterococci* with alpha hemolysis on blood agar medium; (**B**) *Enterococci* on bile esculin agar medium.

Ten of the samples were identified as Gram-positive *cocci* and exhibited alpha hemolytic activity on blood agar medium (Figure 1A). However, only 5 bacterial isolates among the 25 isolates had the ability to grow on bile esculin agar after overnight incubation at 37 °C (Figure 1B). A variety of conventional biochemical tests were performed on the ten *Enterococci* isolates, and the results are shown in Table 2. Our results for the isolation of bacteria showed that 5 isolates among 25 belonged to *Enterococcus* spp., as they were grown on bile esculin. These isolates showed remarkable alpha blood hemolysis, in addition to confirming bacterial identification by 16S rDNA sequencing, which was performed according to previous strategies. *Enterococci* isolates were identified using the VITEK 2 GN analyzer, 2018 modified version. The tested isolates were determined to be *Enterococcus faecalis* with 94% similarity.

### 3.2. Antibiotic Susceptibility

The five *E. faecalis* isolates (EF.1–EF.5) were tested against ten different antibiotics. They exhibited resistance against ten antibiotics (Table 3). Clinical settings are seeing an increase in the number of antibiotic-resistant *E. faecalis* strains, as well as biofilm formation providing protection, making treatments, such as antiseptic rinses or antibacterial dressings, useless.

**Figure 2:**
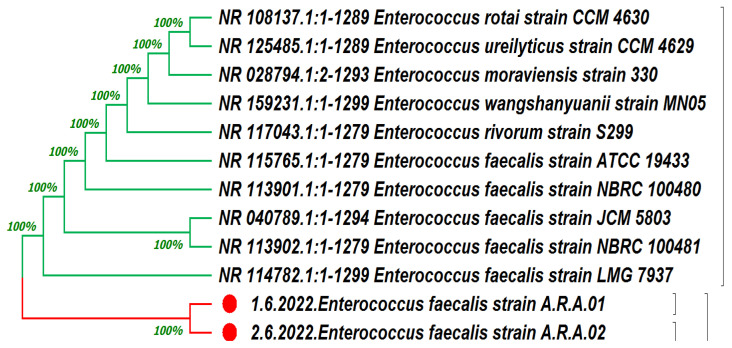
Phylogenetic analysis of the isolated *Enterococcus faecalis* from dental abscesses: *Enterococcus faecalis* A.R.A.01 (ON797462.1) and *Enterococcus faecalis* A.R.A.02 (ON797463.1). Neighbor-joining trees display the phylogenetic position of the isolated and phylogenetically related members of this genus.

Two isolates of *E. faecalis* were used as hosts for the isolation of bacteriophages, as confirmed by the 16s RNA gene sequence. The obtained DNA sequence of the partial 16S RNA gene displayed a high similarity to the *E. faecalis* strain’s 16S ribosomal DNA gene. A phylogenetic tree between these strains and the closely resembled sequences was constructed using MEGA11 software (v11.0.13) (Figure 2).

### 3.3. Genomic Identification of Bacterial Isolates

In the current study, different samples of sewage water were used to isolate two different phages (*E. faecalis*_phage-01 and *E. faecalis*_phage-02) that targeted *E. faecalis*. According to transmission electron microscopy, two phages, *E. faecalis*_phage-01 and *E. faecalis*_phage-02, were morphologically related to the families *Siphoviridae* and *Myoviridae*, respectively.

### 3.4. Phage Isolation and Characterization

**Figure 3:**
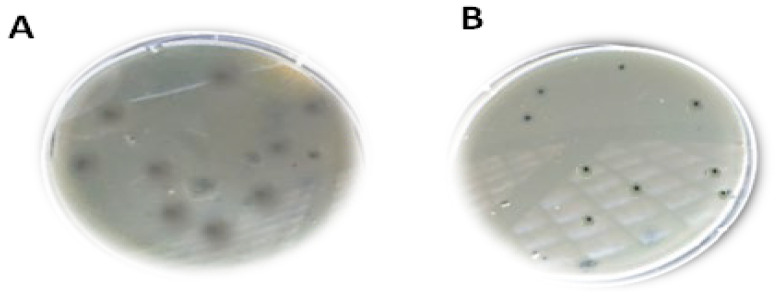
Plaque morphology of *E. faecalis*_phage-01 (**A**) and *E. faecalis*_phage-02 (**B**) after 48 h of culturing on their host lawns.

Two specific phages of *E. faecalis* (*E. faecalis*_phage-01 and *E. faecalis*_phage-02) were obtained from sewage water; they were selected based on their lytic activity by spot testing. They exhibited different plaque morphologies. The titration of phages was conducted using an overlay method and counted at approximately 10^9^ PFU/mL. The dimensions, morphology, and the turbidity of the plaques are described in Figure 3.

**Figure 4:**
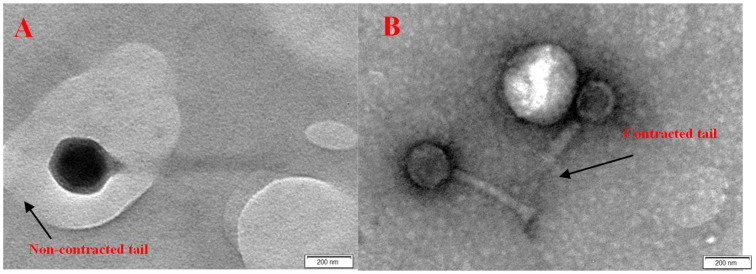
TEM of two isolated *E. faecalis* phages using TEM. (**A**) *E. faecalis*_phage-01, which is related to the family *Siphoviridae*, and (**B**) *E. faecalis*_phage-02, which is a member of the family *Myoviridae*.

Two *E. faecalis* phages (*E. faecalis*_phage-01 and *E. faecalis*_phage-02) targeting dental abscesses were examined via TEM. *E. faecalis*_phage-01 is a member of the family *Siphoviridae*, as revealed by the presence of a long and flexible tail (Figure 4A), and *E. faecalis*_phage-02, which has a contractile tail, is related to the family *Myoviridae* (Figure 4B).

### 3.5. Bioinformatics and In Silico Analysis of Enterococcus Phage

The BLAST analysis results for the RNA ligase sequences of phage-01 and phage-02 revealed a 97.23% similarity to *Enterococcus* phage vB_EfaS_HEf13 (MH618488.1). Additionally, a phylogenetic tree was constructed using a conserved RNA ligase sequence, which included 14 closely related published phages. According to this tree, both phage-01 and phage-02 were classified as belonging to the Enterococcus phage group, specifically associated with the accession number MH618488.1. To further investigate these phages, bioinformatics and in silico tools were employed to annotate and predict the functional and virulence proteins.

**Figure 5:**
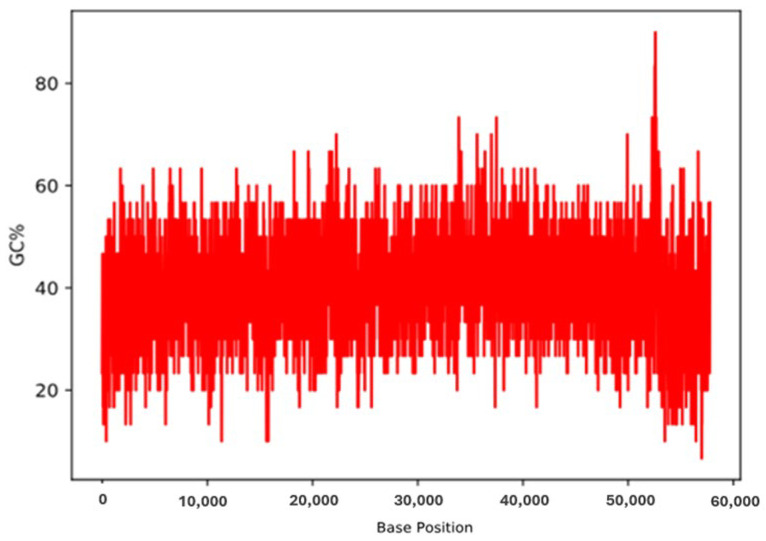
GC content distribution in the genome of *Enterococcus* phage.

**Figure 6:**
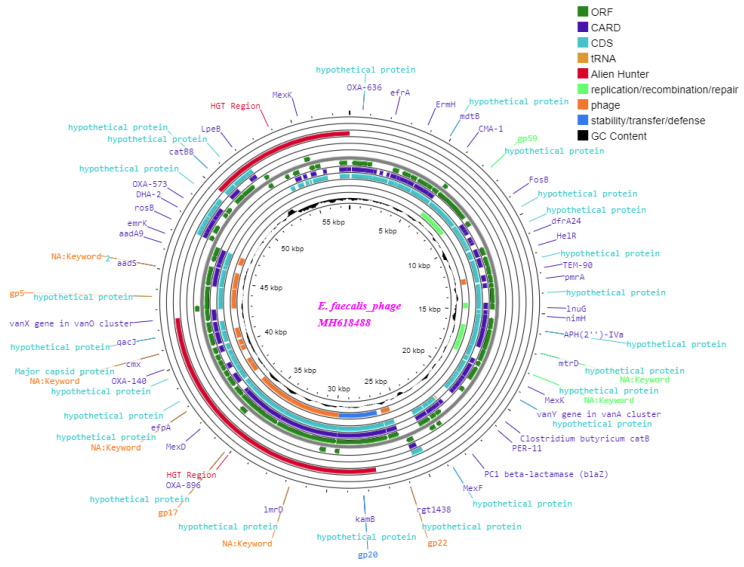
Circular genome map of *Enterococcus* phage constructed using CG View.

The total genome length of *Enterococcus*_phage-01 and phage-02 was determined to be 57.8 kb, with a GC content of 40% (Figure 5). Furthermore, in silico analysis and annotations of the phage genome revealed that a total of 69 open reading frames (ORFs) included 53 located on the indirect strand and 16 on the direct strand (Figure 6). All coding sequences were found to initiate with the ATG start codon. The functional proteins were categorized as follows: (i) Protein families involved in the integration, excision, replication, recombination, repair, stability, defense, or transfer of bacterial mobile genetic elements and phages. Among the predicted ORFs, 19 were associated with mobile orthologous groups. Within these groups, 13 ORFs (68.42%) were predicted to encode proteins involved in phage-related biological processes, such as structural proteins and lysogeny-associated machinery. Additionally, four ORFs (21.05%) were predicted to encode proteins involved in replication, recombination, or repair (RRR), including gp59 and efb37. Two ORFs (10.52%) were anticipated to be associated with the stability, transfer, and defense (STD) of elements from the host machinery or other elements, including gp20 (see Table 4). (ii) Regarding host lysis and adhesion proteins, an analysis using BLASTP on the *Enterococcus* phage genome showed the absence of genes encoding CRISPR-Finder. Additionally, no similarities were found with genes encoding integrase or excisionase. Furthermore, searches against various databases, such as VFDB (Virulence Factor Database), Virulence Finder 2.0, and PAIDB (Pathogenicity Island Database), yielded no results. Conversely, when the *Enterococcus* phage genome was analyzed against the Comprehensive Antibiotic Resistance Database (CARD), a total of 77 comprehensive antibiotic resistance genes were identified. Table S1 summarizes these genes, including their start and end positions, lengths, strands, gene names, drug classes, AMR gene families, and resistance mechanisms.

### 3.6. Host Range Activity

In this study, when assessing the efficacy of the isolated bacteriophage against various bacterial strains, the findings indicated that the bacteriophage’s effectiveness was restricted solely to the strain from which it was isolated. This suggests that the bacteriophage exhibited a high degree of specificity and did not target any bacterial strain other than the one it was isolated from. The two *E. faecalis* lytic phages *E. faecalis*_phage-01 and *E. faecalis*_phage-02 showed high specific activity against *E. faecalis*, but no activity against other genera and species (Table 5).

### 3.7. Genetic Identification of Bacteriophage

**Figure 7:**
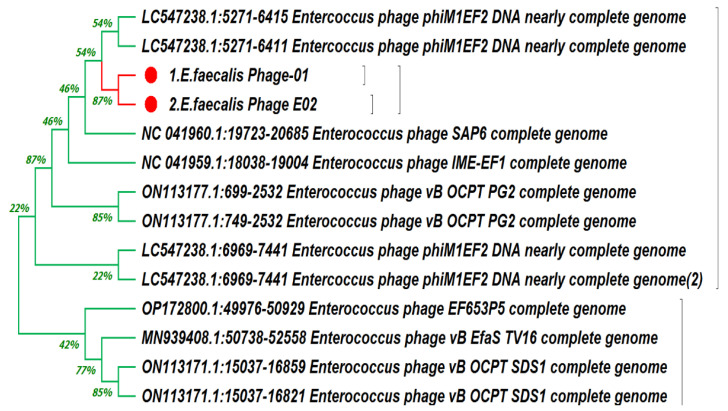
Phylogenetic tree of the *E. faecalis*_phage-01 genome (ON809698) and the *E. faecalis*_phage-02 genome (ON809699).

The two bacteriophages were identified genetically and recorded on GenBank under accession numbers *E. faecalis*_phage-01 genome (ON809698) and *E. faecalis*_phage-02 genome (ON809699). Phylogenetic analyses of the 16S rRNA gene sequences were conducted with MEGA software version 4 (MEGA4). Trees were constructed using the neighbor-joining method based on the maximum composite likelihood model. Phylogenies were also evaluated by the maximum parsimony method and the unweighted pair group method with arithmetic mean (UPGMA) and found to be similar (Figure 7).

### 3.8. One-Step Growth Curve

**Figure 8:**
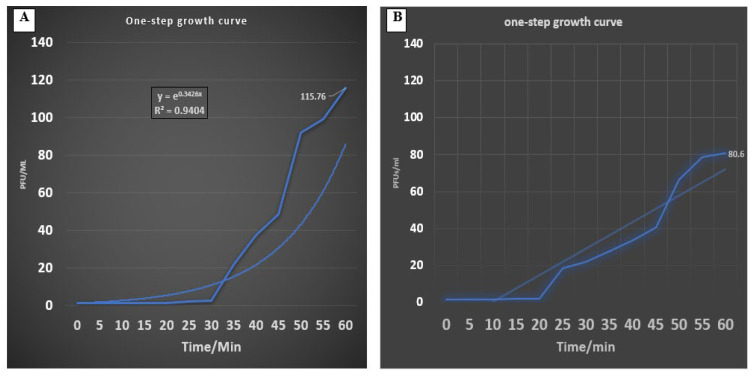
One-step growth curve of (**A**) *E. faecalis*_phage-01 indicating burst size of 86.75 PFU/mL and latent period of 25 min. (**B**) *E. faecalis*_phage-02 indicating burst size of 115.76 PFU/mL and latent period of 30 min.

A one-step growth curve was obtained for each of the two isolated phages (Figure 8). The *Siphoviridae* phage (*E. faecalis*_phage-01) had a burst size of 115.7 PFU/mL with latent periods of 30 min (Figure 8A), and the *Myoviridae* phage (*E. faecalis*_phage-02) had a burst size of 80.6 PFU/mL with a latent period of 25 min (Figure 8B). These results are comparable to those reported in previous studies using *E. faecalis* phages, where the average burst sizes of such phages were approximately 36–122 PFU/mL and the latent periods were 25–50 min.

### 3.9. Thermal and pH Stability

**Figure 9:**
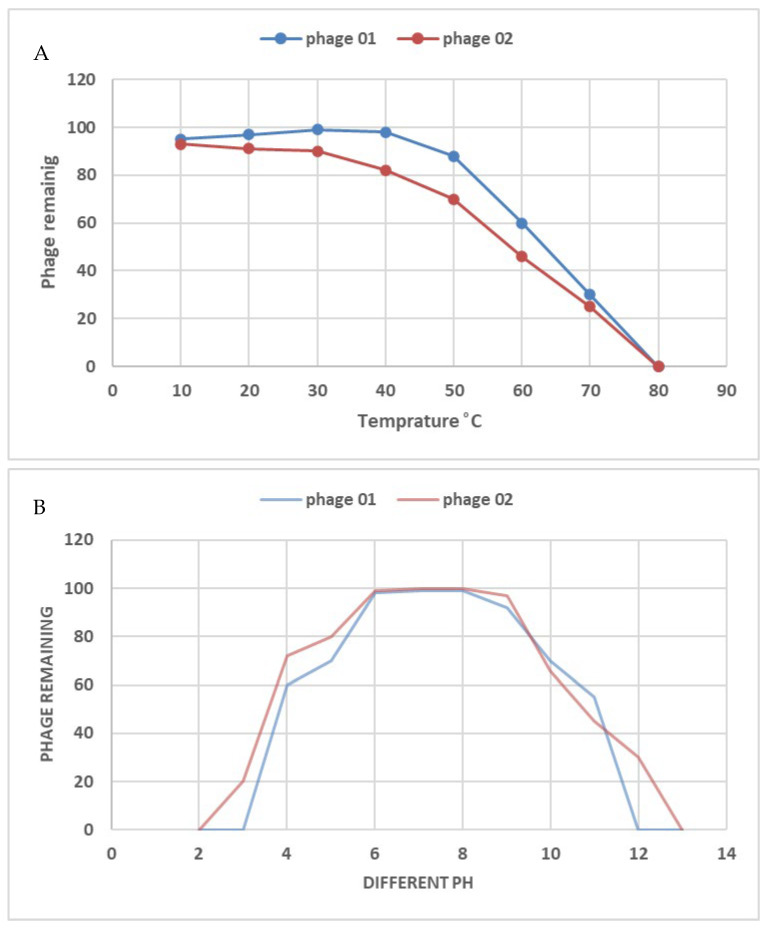
Temperature and pH stability test of two *E. faecalis* phages, *E.faecalis*_phage-01 and *E.faecalis*_phage-02; (**A**) stability of two tested phages at different temperatures, (**B**) stability of two tested phages at different pH. The percentages of phages left over after each treatment are displayed in the data, normalized from the control. Error bars illustrate the variation of values from the mean, as represented by data from three replicates.

The thermal and pH stability of isolated phages were investigated based on residual phage titers after incubation at different pH values and temperatures (Figure 9). Two *E. faecalis* phages were detected in environments with temperatures between 10 and 60 °C. However, after 60 min of heating at 80 °C, no live phages were found. After 60 min at 37 °C, it was discovered that two *E. faecalis* phages were resistant to a pH range of 4–11. The application of phages as biocontrol agents in dental procedures is facilitated by their stability under harsh environmental conditions. Under a temperature range of 10–60 °C and pH range of 4–11, *E. faecalis*_phage-01 and *E. faecalis*_phage-02 were stable. As a result, when used in clinical settings, the isolated phages demonstrated particularly high efficiency when combined with the alkaline disinfectants commonly employed to treat endodontic infections. Regarding the oral epithelial cell lines evaluated in vitro, our isolated phages had no harmful effects and were safe to utilize in prior human research. As a result, using these phages in therapy instead of antibiotics may be promising.

### 3.10. Cytotoxic Effect

**Figure 10:**
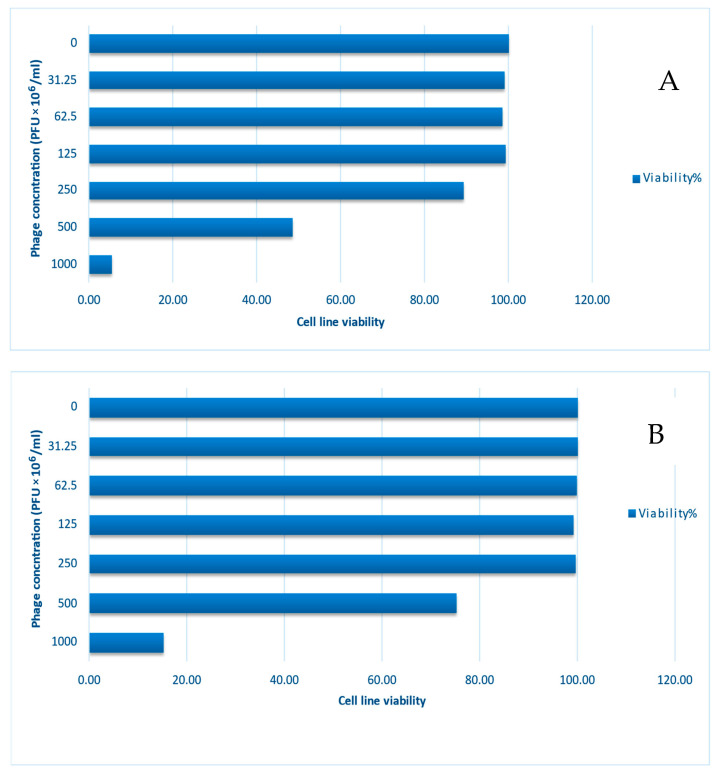
Cytotoxic effect of isolated bacteriophages (**A**) *E. faecalis*_phage-01 and (**B**) *E. faecalis*_phage-02 on oral epithelial cells.

**Figure 11:**
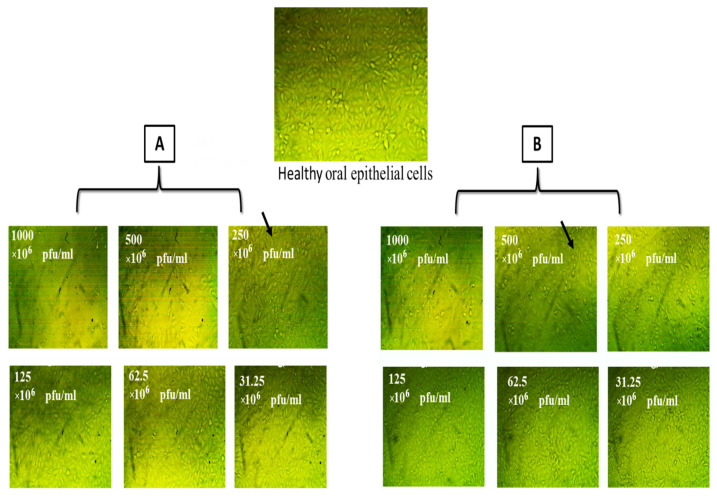
Cytotoxicity of *E. faecalis* bacteriophages (pfu × 10^6^)/mL (**A**) *E. faecalis*_phage-01 (**B**) *E. faecalis*_phage-02 on oral epithelial cells. Where black arrows showed some of available cells within test sample.

Based on our results of the cytotoxic effect of phage solutions on the normal epithelial tissue, which is the abundant type of tissue lining the alimentary canal, especially the oral cavity, isolated phages did not adversely affect oral epithelial cells when used at concentrations of 1000, 500, 250, 125, 62.5, and 31.25 pfu/mL (Table 6, Figure 10 and Figure 11).

## 4. Discussion

The treatment of intraarticular infections is an important component of dental therapy. A frequent opportunistic pathogen that may infect the human oral cavity is *Enterococcus faecalis* [35]. According to Wong et al. [36], in every root canal that was examined, *Enterococcus faecalis* was found. Several studies have demonstrated the importance of using bacteriophages against *E. faecalis* for the treatment of dental problems [33,34,37,38]. In the work by Nasr-Eldin et al. [39], the isolated potential bacteria were identified biochemically, microscopically described using traditional techniques, and validated using the Vitek2 system. Over the past 30 years, antibiotic-resistant *Enterococcus* strains have become increasingly related to nosocomial infections, with increasingly high levels of antibiotic resistance and multidrug resistance [40]. These days, most bacteria can improve their resistance to numerous types of antibiotics. The development of alternative strategies, such as therapy with phages, can play a fundamental role in combating antibiotic-resistant *Enterococcai* infections [41]. In dentistry, bacteriophages appear to be a novel and promising approach to combating resistant intraarticular bacteria, like *E. faecalis*. Phage reduction of the *E. faecalis* biofilm in dental ex vivo models has been demonstrated in several studies. Phage reduction may be paired with standard endodontics, like sodium hypochlorite and EDTA [42]. Furthermore, phage therapy was found to be more effective than antibiotic treatment in models of intraperitoneal and periapical rat infections, as evidenced by the higher survival rates and normal anatomical findings [43].

Our results for the isolation of bacteria showed that 5 isolates among 25 were *Enterococcus* sp. as they had grown on bile esculin. These isolates showed remarkable alpha blood hemolysis. Currently, for bacterial infections, lytic bacteriophages are used to remove multidrug-resistant *E. faecalis* strains and associated biofilms [42,44]. These pathogens were isolated from root canal infections and tested for antibiotic susceptibility by El-Telbany et al. [42]. The two isolated and purified phages in this study, *E. faecalis*_phage-01 and *E. faecalis*_phage-02, were related to the families *Siphoviridae* and *Myoviridae*, respectively. Their genome sequences are compatible with this classification and resemble other phages that have recently been identified [45].

Phage resistance has also been documented in human phage treatment [46], which might pose a challenge to the use of phage therapy. In this way, our research offers important information on how susceptible phage-resistant mutants are to various phages [47]. Some studies suggest that the phage resistance of *E. faecalis* results from mutations in phage receptors, such as the enterococcal polysaccharide antigen (Epa) [48] or the membrane protein PIP [49]. A potential tactic to lower antibiotic dosage and prevent antibiotic resistance during therapy is the combination of phages with antibiotics. Prior research [12,50,51] has demonstrated that administering the antibiotic and phage in turn produced greater results than administering them simultaneously.

The efficacy of phage treatments is determined by the bacterial host range of the phage. It is preferable to limit the host range to a single species, since this minimizes collateral harm and maintains the integrity of the host microbiota [52]. The characterization of phages is made possible by sequencing parts of the genome, which enables their classification as therapeutically helpful. The toxin, virulence, and lysogeny-related genes should be kept out of phages that show promise for phage therapy [53]. A substantial characterization of phages is needed, including complete genomic information, the absence of genes that raise safety concerns, a low risk of lysogenic lifestyle, and stability in reproduction in the production environment [54]. Only a small percentage of all phages have the potential to be used in phage therapy. Only 50% of phages recovered from the environment are thought to be beneficial for therapeutic purposes, according to a recent estimation [55].

Phage therapy has many advantages over conventional therapy through antibiotics. Our two *E. faecalis* phages grew at temperatures ranging from 10 to 60 °C and were resistant to a wide pH range (4–11). Previous research has shown that phages are effective against *E. faecalis* under ranges of 10–50 °C and pH 3–12 [56,57]. Bacteriophages have been used in earlier research to prevent or treat oral infections [58]. Phage isolation is rapid, relatively straightforward, and inexpensive [59]. Phage resistance develops approximately 10-fold slower than antibiotic resistance [59]. Additionally, phages tend to remain effective even under very harsh environmental conditions and have the ability to continue to multiply until the host bacterial population density is greatly reduced [60]. These properties suggest that, unlike conventional chemical antibiotics, phage therapy may require less or more limited dosing while performing as well as or better than conventional therapies. Additionally, most of the phages isolated so far have relatively high host specificity. This advantage of phages diminishes the damaging risk to the human body’s natural microbiota, in addition to eliminating chemical antibiotic side effects [61].

## 5. Conclusions

Two MDR *Enterococcus faecalis* strains, *Enterococcus faecalis A.R.A.01* and *Enterococcus faecalis* A.R.A.02, were isolated and used as hosts for specific phage isolation. Two lytic phages, *E. faecalis*_phage-01 and *E*. *faecalis*_phage-02, were isolated and purified, and found to belong to the families *Siphoviridae* and *Myoviridiae*, as they were described through TEM and genomic identification. They exhibited a high specific host range with *Enterococcus faecalis* among six different bacterial hosts, with latent periods of 30 and 25 min, respectively. They also exhibited stability under wide ranges of pH (4–11) and temperature (10–60 °C), with a low cytotoxic effect on oral epithelial cell lines at different concentrations (1000–31.25 PFU/mL). A promising strategy is being considered to combat biofilms and multidrug-resistant *E. faecalis* strains by conducting treatment with lytic bacteriophages.

## Figures and Tables

**Table 1:** Specific primers used in genomic identification of two *E. faecalis* phages.

Primer	Sequence (5-3)	Length	Tm	GC%	Strand
Forward primer 6	CCATGTGCTGAACGCCTTTT	20	59.6	50	Plus
Reverse primer 6	CTGTGGCGATTGAGCGTTTC	20	60.1	55	Minus
Forward primer 9	AGCTGACTCTTCGCTTGGAG	20	59.7	55	Plus
Reverse primer 9	AAAAGGCGTTCAGCACATGG	20	59.6	50	Minus

**Table 2:** Biochemical characterization of bacterial isolates.

Code Test	EF.1	EF.2	EF.3	EF.4	EF.5
Motility	─	─	─	─	─
Catalase	─	─	─	─	─
Oxidase	─	─	─	─	─
Glucose fermentation	+	+	+	+	+
Nitrate Reduction	+	+	+	+	+
Vogues Proskour	+	+	+	+	+
Indole Production	─	─	─	─	─
Citrate Utilization	─	─	─	─	─
H_2_S Production	─	─	─	─	─
Urease	─	─	─	─	─
Coagulase	─	─	─	─	─

(─) without activity, (+) with remarkable activity, and the five *E. faecalis* isolates (EF.1–EF.5).

**Table 3:** Antibiotic susceptibility test for five *Enterococcus faecalis* isolates against ten different antibiotics.

Test Group	Antimicrobial Agent	Dose	*E. faecalis* Isolates				
			EF.1	EF.2	EF.3	EF.4	EF.5
Glycopeptides	Vancomycin	30 µg	**R**	**R**	**R**	**R**	**R**
Monobactam	Aztreonam	30 µg	**R**	**R**	**R**	**R**	**R**
Cyclic polypeptide	Bacitracin	0.04 µg	**R**	**R**	**R**	**R**	**R**
Lincomycin	Clindamycin	2 µg	**R**	**R**	**R**	**R**	**R**
Fusidane	Fusidic acid	10 µg	**S**	**S**	**S**	**S**	**S**
Carbapenem	Imipenem	10 µg	**R**	**S**	**S**	**S**	**S**
Quinolone	Nalidixic acid	30 µg	**R**	**R**	**R**	**R**	**R**
Fluoroquinolone	Norfloxacin	10 µg	**R**	**R**	**I**	**R**	**I**
	Ofloxacin	5 µg	**I**	**S**	**S**	**S**	**S**
*Aminoglycoside*	Streptomycin	10 µg	**R**	**R**	**R**	**R**	**I**

Where R: resistant, S: sensitive, I: intermediate.

**Table 4:** Functional annotation of mobile OG for *Enterococcus* phage.

No	mobileOG ID	ORF Start	End	Length	Strand	GC%	P_ident	e-Value	Gene Name	Major mobileOG Category
0	**MobileOG_000028221**	**6152**	**8503**	**784**	**−1**	**0.409**	**99.4**	**0**	**gp59**	**Replication/Recombination**
1	**MobileOG_000381065**	**12,559**	**12,993**	**145**	**−1**	**0.423**	**98.6**	**4.80 × 10^−74^**	**gp48**	**Phage**
2	**MobileOG_000385144**	**14,480**	**14,920**	**147**	**−1**	**0.363**	**98.6**	**5.9 × 10^−80^**	**efb37**	**Replication/Recombination**
3	**MobileOG_000766253**	**15,455**	**16,207**	**251**	**−1**	**0.335**	**99.6**	**1.70 × 10^−143^**	**NA:Keyword**	**Stability/Transfer/Defense**
4	**MobileOG_000766252**	**16,220**	**17,584**	**455**	**−1**	**0.415**	**100**	**1.60 × 10^−256^**	**NA:Keyword**	**Replication/Recombination**
5	**MobileOG_000762900**	**17,596**	**18,372**	**259**	**−1**	**0.405**	**100**	**6.80 × 10^−148^**	**NA:Keyword**	**Replication/Recombination**
6	**MobileOG_000028219**	**25,673**	**26,386**	**238**	**−1**	**0.445**	**99.6**	**3.90 × 10^−142^**	**gp22**	**Phage/Lysis/Lysogeny**
7	**MobileOG_000028218**	**26,734**	**29,760**	**1009**	**−1**	**0.403**	**96.7**	**0**	**gp20**	**Stability/Transfer/Defense**
8	**MobileOG_000773315**	**29,773**	**33,765**	**1331**	**−1**	**0.421**	**99.5**	**0**	**NA:Keyword**	**Phage**
9	**MobileOG_000028217**	**33,779**	**36,664**	**962**	**−1**	**0.45**	**98.8**	**0**	**gp17**	**Phage/Structural**
10	**MobileOG_000762915**	**37,496**	**38,185**	**230**	**−1**	**0.439**	**100**	**9.10 × 10^−128^**	**NA:Keyword**	**Phage**
11	**MobileOG_000763662**	**38,206**	**38,640**	**145**	**−1**	**0.411**	**99.3**	**1.40 × 10^−78^**	**NA:Keyword**	**Phage**
12	**MobileOG_000762916**	**39,411**	**39,815**	**135**	**−1**	**0.44**	**97.8**	**7.80 × 10^−71^**	**NA:Keyword**	**Phage**
13	**MobileOG_000766248**	**39,875**	**40,315**	**147**	**−1**	**0.44**	**98.6**	**7.20 × 10^−70^**	**NA:Keyword**	**Phage**
14	**MobileOG_000763661**	**40,470**	**41,276**	**269**	**−1**	**0.408**	**98.9**	**4.30 × 10^−145^**	**NA:Keyword**	**Phage**
15	**MobileOG_000773312**	**42,110**	**42,865**	**252**	**−1**	**0.43**	**99.6**	**1.50 × 10^−138^**	**NA:Keyword**	**Phage**
16	**MobileOG_000381009**	**42,877**	**44,412**	**512**	**−1**	**0.411**	**99.8**	**4.10 × 10^−290^**	**gp5**	**Phage/Structural**
17	**MobileOG_000766245**	**44,469**	**45,740**	**424**	**−1**	**0.424**	**100**	**8.30 × 10^−252^**	**NA:Keyword**	**Phage**
18	**MobileOG_000768258**	**46,428**	**47,027**	**200**	**−1**	**0.397**	**91**	**2.10 × 10^−96^**	**NA:Keyword**	**Phage**

**Table 5:** Phage activity against different bacterial genera and species.

Bacterial Species	*E. faecalis*_phage-01	*E. faecalis*_phage-02
*E. faecalis*	+	+
*S. mutans*	−	−
*E. faecium*	−	−
*S. aureus*	−	−
*E. coli*	−	−
*P. aeruginosa*	−	−

(+) remarkable phage activity, (−) no phage activity.

**Table 6:** Cytotoxic effect of isolated Bacteriophages *E. faecalis*_phage-01 and *E. faecalis*_phage-02 on oral epithelial cells.

ID	µg/mL	O.D			Mean O.D	±SE	Viability %	Toxicity %	IC50 ± SD
OEC	--------	0.622	0.625	0.619	0.622	0.001732	100	0	µg
***E. faecalis*** **_phage-01**	1000	0.032	0.038	0.033	0.034333	0.001856	5.51982851	94.48017149	563.46 ± 4.93
	500	0.317	0.287	0.302	0.302	0.00866	48.55305466	51.44694534	
	250	0.547	0.557	0.562	0.555333	0.00441	89.28188639	10.71811361	
	125	0.62	0.617	0.617	0.618	0.001	99.35691318	0.643086817	
	62.5	0.609	0.621	0.611	0.613667	0.003712	98.6602358	1.339764202	
	31.25	0.608	0.624	0.619	0.617	0.004726	99.19614148	0.803858521	
***E. faecalis*** **_phage-02**	1000	0.081	0.108	0.093	0.094	0.00781	15.11254019	84.88745981	700 ± 8.59
	500	0.46	0.473	0.469	0.467333	0.003844	75.13397642	24.86602358	
	250	0.622	0.617	0.619	0.619333	0.001453	99.57127546	0.428724544	
	125	0.608	0.624	0.62	0.617333	0.004807	99.24973205	0.750267953	
	62.5	0.619	0.623	0.621	0.621	0.001155	99.8392283	0.160771704	
	31.25	0.625	0.62	0.621	0.622	0.001528	100	0	

## Data Availability

All authors declare that the data supporting the findings of this study are available within the article.

## Supplementary Materials

The following supporting information can be downloaded at: https://www.mdpi.com/article/10.3390/medicina60030501/s1, Table S1: Functional annotation of comprehensive antibiotic resistance genes for *Enterococcus*_phage.

## References

[1] Stephens M.B., Wiedemer J.P., Kushner G.M. (2018). Dental problems in primary care. Am. Fam. Physician.

[2] Roberts R.M., Hersh A.L., Shapiro D.J., Fleming-Dutra K.E., Hicks L.A.J. (2019). Antibiotic prescriptions associated with dental-related emergency department visits. Ann. Emerg. Med..

[3] Hailu F.A., Hailu Y.A., Hailu T.A. (2020). Dental anatomy and physiology of human tooth and the consequences of pathogenic microbiota on the oral cavity. J. Clin. Case Stud. Rev. Rep..

[4] Shakya M., Kayastha P.K., Jiao H. (2018). Oral flora: Protection or destruction of dental tissue. Int. J. Endorsing Health Sci. Res. (IJEHSR).

[5] Schmidt J., Kunderova M., Pilbauerova N., Kapitan M. (2021). A review of evidence-based recommendations for pericoronitis management and a systematic review of antibiotic prescribing for pericoronitis among dentists: Inappropriate pericoronitis treatment is a critical factor of antibiotic overuse in dentistry. Int. J. Environ. Res. Public Health.

[6] Arhun N., Arman-Özçırpıcı A., Çehreli S.B., Gülşahı K., Özsoy Ö.P. (2023). The Restorative Dentist and Orthodontist: Orthodontic Implications of Dental Caries, Tooth Fracture, Exposed Dental Pulp, and Esthetic Improvements. Integr. Clin. Orthod..

[7] Thompson W., McEachan R., Pavitt S., Douglas G., Bowman M., Boards J., Sandoe J. (2020). Clinician and patient factors influencing treatment decisions: Ethnographic study of antibiotic prescribing and operative procedures in out-of-hours and general dental practices. Antibiotics.

[8] Zaken H.B., Kraitman R., Coppenhagen-Glazer S., Khalifa L., Alkalay-Oren S., Ben-Gal G., Beyth N., Hazan R. (2021). Isolation and characterization of anti-Streptococcus mutans phage as a possible treatment agent for caries. Viruses.

[9] Ayesha F. (2020). Polymicrobial Interactions between Selected Microbes of Oral Biofilm and Differential Expression of ALS Genes and Their Related Proteins/Ayesha Fahim. Doctoral Dissertation.

[10] Geraldes C., Tavares L., Gil S., Oliveira M. (2022). Enterococcus Virulence and Resistant Traits Associated with Its Permanence in the Hospital Environment. Antibiotics.

[11] García-Solache M., Rice L.B. (2019). The Enterococcus: A model of adaptability to its environment. Clin. Microbiol. Rev..

[12] Liu C., Hong Q., Chang R.Y.K., Kwok P.C.L., Chan H.-K. (2022). Phage–Antibiotic therapy as a promising strategy to combat multidrug-resistant infections and to enhance antimicrobial efficiency. Antibiotics.

[13] Federici S., Nobs S.P., Elinav E. (2021). Phages and their potential to modulate the microbiome and immunity. Cell. Mol. Immunol..

[14] Marantos A. (2023). Population Dynamics of Phage-Bacteria Ecosystems under Challenging Conditions. Ph.D. Thesis.

[15] Liu D., Van Belleghem J.D., de Vries C.R., Burgener E., Chen Q., Manasherob R., Aronson J.R., Amanatullah D.F., Tamma P.D., Suh G.A. (2021). The safety and toxicity of phage therapy: A review of animal and clinical studies. Viruses.

[16] Ssekatawa K., Byarugaba D.K., Kato C.D., Wampande E.M., Ejobi F., Tweyongyere R., Nakavuma J.L. (2021). A review of phage mediated antibacterial applications. Alex. J. Med..

[17] Łobocka M., Dąbrowska K., Górski A. (2021). Engineered bacteriophage therapeutics: Rationale, challenges and future. BioDrugs.

[18] Abdelsattar A., Dawoud A., Makky S., Nofal R., Aziz R.K., El-Shibiny A. (2022). Bacteriophages: From isolation to application. Curr. Pharm. Biotechnol..

[19] Chung K.M., Nang S.C., Tang S. (2023). The Safety of Bacteriophages in Treatment of Diseases Caused by Multidrug-Resistant Bacteria. Pharmaceuticals.

[20] Miller J.M., Miller S.A. (2017). A Guide to Specimen Management in Clinical Microbiology.

[21] Gupta A., Shah A.A., Khursheed S., Rashid A., Kumar V. (2022). Isolation, identification, speciation, and antibiogram of enterococcus species by conventional methods and assessment of the prevalence of vana genotype among VRE. J. Med. Pharm. Allied. Sci..

[22] Alhamadani Y.S.T., Oudah A.S. (2022). Study of the Bacterial Sensitivity to different Antibiotics which are isolated from patients with UTI using Kirby-Bauer Method. J. Biomed. Biochem..

[23] Asbell P.A., Sanfilippo C.M., Sahm D.F., DeCory H.H. (2020). Trends in antibiotic resistance among ocular microorganisms in the United States from 2009 to 2018. JAMA Ophthalmol..

[24] Alsamman A.M., Khedr M., Kabary H.A., El-Sehrawy M. (2023). Elimination of pathogenic multidrug resistant isolates through different metal oxide nanoparticles synthesized from organic plant and microbial sources. Microb. Pathog..

[25] Church D.L., Cerutti L., Gürtler A., Griener T., Zelazny A., Emler S. (2020). Performance and application of 16S rRNA gene cycle sequencing for routine identification of bacteria in the clinical microbiology laboratory. Clin. Microbiol. Rev..

[26] Thung T., Siti Norshafawatie B., Premarathne J., Chang W., Loo Y., Kuan C., New C., Ubong A., Ramzi O., Mahyudin N.A. (2017). Isolation of food-borne pathogen bacteriophages from retail food and environmental sewage. Int. Food Res. J..

[27] Kifelew L.G., Warner M.S., Morales S., Thomas N., Gordon D.L., Mitchell J.G., Speck P.G. (2020). Efficacy of lytic phage cocktails on Staphylococcus aureus and Pseudomonas aeruginosa in mixed-species planktonic cultures and biofilms. Viruses.

[28] Nguyen L.V. (2020). Evaluation of Variables Affecting the Success of Typing Shed Dog Hair Using Direct PCR.

[29] Andrade-Martínez J.S., Camelo Valera L.C., Chica Cardenas L.A., Forero-Junco L., López-Leal G., Moreno-Gallego J.L., Rangel-Pineros G., Reyes A.J.M., Reviews M.B. (2022). Computational tools for the analysis of uncultivated phage genomes. Microbiol. Mol. Biol. Rev..

[30] Alcock B.P., Raphenya A.R., Lau T.T., Tsang K.K., Bouchard M., Edalatmand A., Huynh W., Nguyen A.-L.V., Cheng A.A., Liu S. (2020). CARD 2020: Antibiotic resistome surveillance with the comprehensive antibiotic resistance database. Nucleic Acids Res..

[31] Kumar S., Stecher G., Li M., Knyaz C., Tamura K. (2018). MEGA X: Molecular evolutionary genetics analysis across computing platforms. Mol. Biol. Evol..

[32] Kropinski A.M., Mazzocco A., Waddell T.E., Lingohr E., Johnson R.P. (2009). Enumeration of bacteriophages by double agar overlay plaque assay. Bacteriophages; Methods in Molecular Biology.

[33] Wang R., Xing S., Zhao F., Li P., Mi Z., Shi T., Liu H., Tong Y. (2018). Characterization and genome analysis of novel phage vB_EfaP_IME195 infecting Enterococcus faecalis. Virus Genes.

[34] Mekky A.E., Emam A.E., Selim M.N., Abdelmouty E.S., Khedr M. (2023). Antibacterial and antineoplastic MCF-7 and HePG-2 characteristics of the methanolic (80%) clove (*Syzygium aromaticum* L.) extract. Biomass Convers. Biorefinery.

[35] Najafi K., Ganbarov K., Gholizadeh P., Tanomand A., Rezaee M.A., Mahmood S.S., Asgharzadeh M., Kafil H.S. (2020). Oral cavity infection by Enterococcus faecalis: Virulence factors and pathogenesis. Rev. Res. Med. Microbiol..

[36] Wong J., Manoil D., Näsman P., Belibasakis G.N., Neelakantan P. (2021). Microbiological aspects of root canal infections and disinfection strategies: An update review on the current knowledge and challenges. Front. Oral Health.

[37] Hyman P. (2019). Phages for phage therapy: Isolation, characterization, and host range breadth. Pharmaceuticals.

[38] Xing S., Zhang X., Sun Q., Wang J., Mi Z., Pei G., Huang Y., An X., Fu K., Zhou L. (2017). Complete genome sequence of a novel, virulent Ahjdlikevirus bacteriophage that infects Enterococcus faecium. Arch. Virol..

[39] Nasr-Eldin M.A., El-Dougdoug N.K., Elazab Y.H., Esmael A. (2021). Isolation and Characterization of Two Virulent Phages to Combat Staphylococcus aureus and Enterococcus faecalis causing Dental Caries. J. Pure Appl. Microbiol..

[40] Esmael A., Hassan M.G., Amer M.M., Abdelrahman S., Hamed A.M., Abd-Raboh H.A., Foda M.F. (2020). Antimicrobial activity of certain natural-based plant oils against the antibiotic-resistant acne bacteria. Saudi J. Biol. Sci..

[41] Esmat M.M., Abdelhamid A.G., Abo-ELmaaty S.A., Nasr-Eldin M.A., Hassan M.G., Khattab A.A., Esmael A. (2017). Antibiotics and phage sensitivity as interventions for controlling Escherichia coli isolated from clinical specimens. J. Pure Appl. Microbiol..

[42] El-Telbany M., El-Didamony G., Askora A., Ariny E., Abdallah D., Connerton I.F., El-Shibiny A. (2021). Bacteriophages to control multi-drug resistant Enterococcus faecalis infection of dental root canals. Microorganisms.

[43] Xiang Y., Ma C., Yin S., Song F., Qin K., Ding Y., Yang X., Song P., Ji X., Wei Y. (2022). Phage therapy for refractory periapical periodontitis caused by Enterococcus faecalis in vitro and in vivo. Appl. Microbiol. Biotechnol..

[44] Bhardwaj S.B., Mehta M., Sood S., Sharma J. (2020). Isolation of a novel phage and targeting biofilms of drug-resistant oral enterococci. J. Glob. Infect. Dis..

[45] Dion M.B., Oechslin F., Moineau S. (2020). Phage diversity, genomics and phylogeny. Nat. Rev. Microbiol..

[46] Pires D.P., Costa A.R., Pinto G., Meneses L., Azeredo J. (2020). Current challenges and future opportunities of phage therapy. FEMS Microbiol. Rev..

[47] McGee L.W., Barhoush Y., Shima R., Hennessy M. (2023). Phage-resistant mutations impact bacteria susceptibility to future phage infections and antibiotic response. Ecol. Evol..

[48] Canfield G.S., Chatterjee A., Espinosa J., Mangalea M.R., Sheriff E.K., Keidan M., McBride S.W., McCollister B.D., Hang H.C., Duerkop B.A. (2021). Lytic bacteriophages facilitate antibiotic sensitization of *Enterococcus faecium*. Antimicrob. Agents Chemother..

[49] Liu J., Zhu Y., Li Y., Lu Y., Xiong K., Zhong Q., Wang J. (2022). Bacteriophage-Resistant Mutant of Enterococcus faecalis Is Impaired in Biofilm Formation. Front. Microbiol..

[50] Li X., He Y., Wang Z., Wei J., Hu T., Si J., Tao G., Zhang L., Xie L., Abdalla A.E. (2021). A combination therapy of Phages and Antibiotics: Two is better than one. Int. J. Biol. Sci..

[51] Morrisette T., Kebriaei R., Lev K.L., Morales S., Rybak M.J. (2020). Bacteriophage therapeutics: A primer for clinicians on phage-antibiotic combinations. Pharmacother. J. Hum. Pharmacol. Drug Ther..

[52] Lauman P., Dennis J.J. (2021). Advances in phage therapy: Targeting the Burkholderia cepacia complex. Viruses.

[53] Xue J., Mirzaei M.K., Costa R., Smith S., Tiamani K., Ma T., Deng L. (2024). Human virome in health and disease. Molecular Medical Microbiology.

[54] Abril A.G., Carrera M., Notario V., Sánchez-Pérez Á., Villa T.G. (2022). The use of bacteriophages in biotechnology and recent insights into proteomics. Antibiotics.

[55] Lin J., Du F., Long M., Li P. (2022). Limitations of phage therapy and corresponding optimization strategies: A review. Molecules.

[56] Alghamdi F., Shakir M. (2020). The influence of Enterococcus faecalis as a dental root canal pathogen on endodontic treatment: A systematic review. Cureus.

[57] Lee D., Im J., Na H., Ryu S., Yun C.-H., Han S.H. (2019). The novel Enterococcus phage vB_EfaS_HEf13 has broad lytic activity against clinical isolates of Enterococcus faecalis. Front. Microbiol..

[58] Shlezinger M., Khalifa L., Houri-Haddad Y., Coppenhagen-Glazer S., Resch G., Que Y.-A., Beyth S., Dorfman E., Hazan R., Beyth N. (2017). Phage therapy: A new horizon in the antibacterial treatment of oral pathogens. Curr. Top. Med. Chem..

[59] Figueiredo C.M., Malvezzi Karwowski M.S., da Silva Ramos R.C.P., de Oliveira N.S., Peña L.C., Carneiro E., Freitas de Macedo R.E., Rosa E.A.R. (2021). Bacteriophages as tools for biofilm biocontrol in different fields. Biofouling.

[60] Maat V.D. (2022). Adaptations of Hospital-Acquired Enterococcus Faecium. Ph.D. Thesis.

[61] Fernández L., Duarte A.C., Rodríguez A., García P. (2021). The relationship between the phageome and human health: Are bacteriophages beneficial or harmful microbes?. Benef. Microbes.

