# Supplementary material for: Fully Characterized Effective Bacteriophages Specific against Antibiotic-Resistant Enterococcus faecalis, the Causative Agent of Dental Abscess
Source: Medicina (Kaunas). 2024 Mar 19;60(3):501. doi: 10.3390/medicina60030501 (PMC10972216; doi:10.3390/medicina60030501)
Supplement: Supplementary file 1 [file medicina-60-00501-s001.zip › medicina-2881612-supplementary.pdf]

Supplementary file

Table S1: Functional annotation of comprehensive antibiotic resistance genes for *Enterococcus*_phage

Contig	Start	Stop	(+/-)	Pass Bit score	Best Hit Bit score	Best Hit ARO	Best Identities	ARO id	Drug Class	Resistance Mechanism	AMR Gene Family
**MH618488.1_1**	3	101	-	275	23.8682	**AAC(6')-Ic**	40	3002549	Aminoglycoside	Inactivation	AAC(6')
**MH618488.1_2**	148	1110	-	500	25.0238	**OXA-636**	27.78	3005857	Carbapenem; cephalosporin	Inactivation	OXA beta-lactamase
**MH618488.1_3**	1112	1378	-	960	22.3274	**efrA**	100	3003948	Fluoroquinolone; rifamycin	Antibiotic efflux	ATP-binding cassette (ABC) antibiotic efflux
**MH618488.1_4**	1371	1619	-	600	22.7126	**emtA**	38.89	3004669	Lincosamide; orthosomycin	Target alteration	Non-erm 23S ribosomal RNA methyltransferase
**MH618488.1_5**	1633	1842	-	700	23.8682	**OCH-3**	42.86	3002516	Monobactam; cephamycin	Inactivation	OCH beta-lactamase
**MH618488.1_7**	2051	2275	-	960	24.2534	**efrA**	30.19	3003948	Macrolide fluoroquinolone	Antibiotic efflux	ATP-binding cassette (ABC) efflux pump
**MH618488.1_9**	2532	2726	-	900	24.2534	**otr(B)**	31.43	3002892	Tetracycline	Antibiotic efflux	Major facilitator superfamily (MFS)
**MH618488.1_10**	2805	3236	-	500	25.7942	**NDM-10**	27.59	3002360	Carbapenem; cephalosporin;	Inactivation	NDM beta-lactamase
**MH618488.1_11**	3249	3449	-	275	22.3274	**AAC (6')-Iy**	33.33	3002569	Aminoglycoside	Inactivation	AAC(6')
**MH618488.1_12**	3460	4044	-	500	24.2534	**ErmH**	47.06	3002823	Lincosamide streptogramin	Target alteration	Erm 23S ribosomal RNA methyltransferase
**MH618488.1_13**	4045	4503	-	500	24.2534	**OXA-25**	42.86	3001420	Carbapenem; cephalosporin	Inactivation	OXA beta-lactamase
**MH618488.1_14**	4562	5257	-	1800	25.7942	**mdtB**	33.33	3000793	Aminocoumarin	Antibiotic efflux	Resistance-nodulation-cell division (RND)
**MH618488.1_15**	5250	5642	-	600	26.9498	**CfxA6**	33.33	3003097	Cephamycin	Inactivation	CfxA beta-lactamase
**MH618488.1_16**	5644	6078	-	700	25.409	**CMA-1**	18.18	3006922	Cephalosporin	Inactivation	CMA beta-lactamase
**MH618488.1_19**	8767	9144	-	225	25.7942	**FosB**	29.41	3000172	Phosphonic acid antibiotic	Inactivation	Fosfomycin thiol transferase
**MH618488.1_20**	9145	9354	-	600	21.9422	**PRC-1**	25	3007085	Cephalosporin	Inactivation	PRC beta-lactamase

**MH618488.1_21**	9357	9566	-	500	24.6386	**cfrC**	34.62	3004146	Streptogramin oxazolidinone	Target alteration	Cfr 23S ribosomal RNA methyltransferase
**MH618488.1_23**	9802	9996	-	1900	22.3274	**AcrF**	30	3000502	Fluoroquinolone; cephalosporin;	antibiotic efflux	Resistance-nodulation-cell division (RND)
**MH618488.1_24**	9997	10176	-	700	24.2534	**CMA-2**	36.11	3006923	Cephalosporin	inactivation	CMA beta-lactamase
**MH618488.1_25**	10189	10362	-	900	28.8758	**emrY**	35.29	3000254	Tetracycline antibiotic	Antibiotic efflux	Major facilitator superfamily (MFS) efflux pump
**MH618488.1_27**	10713	11276	-	300	25.7942	**dfrA24**	33.33	3002856	Diaminopyrimidine	Target replacement	Trimethoprim resistant dihydrofolate reductase dfr
**MH618488.1_28**	11368	12000	-	1000	26.1794	**HelR**	30	3007208	Rifamycin antibiotic	Target protection	Helicase-like RNA polymerase protection protein
**MH618488.1_30**	12559	12993	-	500	25.7942	**TEM-90**	30.3	3000957	Monobactam; cephalosporin;	Inactivation	TEM beta-lactamase
**MH618488.1_31**	13130	13459	-	750	23.483	**pmrA**	40	3000822	Fluoroquinolone	Antibiotic efflux	Major facilitator superfamily (MFS) antibiotic efflux pump
**MH618488.1_33**	14480	14920	-	450	27.335	**lnuG**	35.59	3004085	Lincosamide	Inactivation	Lincosamide nucleotidyltransferase (LNU)
**MH618488.1_34**	14993	15253	-	150	22.7126	**nimH**	26.92	3007110	Nitroimidazole	Inactivation	Nitroimidazole reductase
**MH618488.1_35**	15277	15489	-	810	23.0978	**cdeA**	40.74	3003835	Fluoroquinolone	Antibiotic efflux	Multidrug and toxic compound extrusion (MATE) transporter
**MH618488.1_36**	15455	16207	-	550	25.409	**APH(2")-IVa**	28.21	3002637	Aminoglycoside antibiotic	Inactivation	APH (2")
**MH618488.1_37**	16220	17584	-	2130	26.1794	**mtrD**	27.66	3000811	Macrolide antibiotic; penam	Antibiotic efflux	Resistance-nodulation-cell division (RND) antibiotic efflux pump

**MH618488.1_38**	17596	18372	-	2200	28.1054	**Iles resistance to mupirocin**	30.23	3003730	Mupirocin-like antibiotic	Target alteration	Antibiotic-resistant isoleucyl-tRNA synthetase (ileS)
**MH618488.1_39**	18421	18774	-	1900	24.2534	**MexK**	40.91	3003693	Macrolide; tetracycline	Antibiotic efflux	Resistance-nodulation-cell division (RND)
**MH618488.1_40**	18849	19793	-	50	25.0238	**vanY gene in vanA cluster**	37.78	3002955	Glycopeptide	Target alteration	VanY; glycopeptide resistance gene
**MH618488.1_41**	19823	20035	-	700	25.0238	**vanS gene in vanM cluster**	34.21	3002939	Glycopeptide	Target alteration	VanS; glycopeptide resistance gene
**MH618488.1_42**	20047	20235	-	300	24.2534	**tet[35]**	43.75	3000556	Tetracycline	Target protection	Tetracycline-resistant ribosomal protection protein
**MH618488.1_43**	20249	20401	-	880	21.1718	**arlS**	42.86	3000839	Fluoroquinolone	Antibiotic efflux	Major facilitator superfamily (MFS) antibiotic efflux pump
**MH618488.1_44**	20401	20733	-	400	24.6386	**C.butyricum catB**	25.86	3002674	Phenicol antibiotic	Inactivation	Chloramphenicol acetyltransferase (CAT)
**MH618488.1_45**	20733	20984	-	300	24.2534	**AAC[36]-Ia**	52.63	3002528	Aminoglycoside	Inactivation	AAC [36]
**MH618488.1_46**	20984	21229	-	500	24.6386	**PER-11**	25	3006132	Cephalosporin	Inactivation	PER beta-lactamase
**MH618488.1_47**	22038	22313	-	1200	24.2534	**TaeA**	39.29	3003986	Pleuromutilin antibiotic	Antibiotic efflux	ATP-binding cassette (ABC) antibiotic efflux pump
**MH618488.1_48**	22348	22548	-	750	24.6386	**bmr**	25.45	3003007	Fluoroquinolone	Antibiotic efflux	Major facilitator superfamily (MFS) antibiotic efflux pump
**MH618488.1_49**	22548	22934	-	500	24.6386	**PC1 beta-lactamase**	35.48	3000621	Penam	Inactivation	BlaZ beta-lactamase
**MH618488.1_50**	22937	23371	-	980	24.6386	**opcM**	30.23	3003037	Fluoroquinolone aminoglycoside	Antibiotic efflux	Resistance-nodulation-cell division (RND)

**MH618488.1_51**	23424	24248	-	2110	25.0238	**MexF**	40	3000804	Fluoroquinolone diaminopyrimidine	Antibiotic efflux	Resistance-nodulation-cell division (RND)
**MH618488.1_53**	24961	25389	+	500	26.1794	**CARB-6**	50	3002245	Penam	Inactivation	CARB beta-lactamase
**MH618488.1_54**	25673	26386	-	600	30.0314	**rgt1438**	29.31	3002883	Rifamycin antibiotic	Inactivation	Rifampin glycosyltransferase
**MH618488.1_55**	26433	26786	-	500	23.8682	**OXA-85**	28.95	3001780	Carbapenem; cephalosporin;	Inactivation	OXA beta-lactamase
**MH618488.1_56**	26734	29760	-	400	33.4982	**kamB**	26.42	3004102	Aminoglycoside	Target alteration	16S rRNA methyltransferase (A1408)
**MH618488.1_57**	29773	33765	-	1320	27.7202	**lmrD**	41.67	3002882	Lincosamide	Antibiotic efflux	ATP-binding cassette (ABC) antibiotic efflux pump
**MH618488.1_58**	33779	36664	-	500	26.9498	**OXA-896**	31.15	3006087	carbapenem	Inactivation	OXA beta-lactamase
**MH618488.1_59**	36677	36901	-	1800	25.7942	**MexD**	27.66	3000801	Fluoroquinolone; diaminopyrimidine	Antibiotic efflux	Resistance-nodulation-cell division (RND)
**MH618488.1_61**	37496	38185	-	1000	25.409	**efpA**	44.44	3003955	rifamycin	Antibiotic efflux	Major facilitator superfamily (MFS) antibiotic efflux pump
**MH618488.1_63**	38653	39033	-	200	23.8682	**vanT gene in vanE cluster**	30.3	3002971	Glycopeptide	Target alteration	Glycopeptide resistance gene; vanT
**MH618488.1_66**	39875	40315	-	420	23.8682	**OXA-140**	30.38	3001452	Carbapenem; cephalosporin;	Inactivation	OXA beta-lactamase
**MH618488.1_67**	40470	41276	-	700	26.9498	**cmx**	41.86	3002703	Phenicol antibiotic	Antibiotic efflux	Major facilitator superfamily (MFS) antibiotic efflux pump
**MH618488.1_68**	41325	41999	-	75	26.1794	**qacJ**	35.71	3007014	Disinfecting agents and antiseptics	Antibiotic efflux	Small multidrug resistance [37] antibiotic efflux pump
**MH618488.1_69**	42110	42865	-	200	26.1794	**vanX gene in vanO cluster**	41.67	3002954	Glycopeptide antibiotic	Target alteration	VanX; Glycopeptide

											resistance gene cluster
**MH618488.1_70**	42877	44412	-	375	26.9498	**AcrAB-TolC**	34.04	3003807	Fluoroquinolone; tetracycline	Target alteration;	Resistance-nodulation-cell division
**MH618488.1_71**	44469	45740	-	500	27.7202	**aadS**	23.85	3004683	Aminoglycoside	Inactivation	ANT(6)
**MH618488.1_73**	46070	46450	-	450	25.0238	**aadA9**	30	3002609	Aminoglycoside	Inactivation	ANT(3")
**MH618488.1_74**	46428	47027	-	600	26.5646	**emrK**	47.83	3000206	Tetracycline	Antibiotic efflux	Major facilitator superfamily (MFS)
**MH618488.1_75**	47308	47622	+	1080	23.483	**rosB**	42.11	3003049	Peptide antibiotic	Antibiotic efflux	Major facilitator superfamily (MFS)
**MH618488.1_76**	47622	47879	+	500	22.3274	**APH (6)-Id**	32.65	3002660	Aminoglycoside	Inactivation	APH(6)
**MH618488.1_77**	47881	48324	+	700	25.7942	**DHA-2**	55	3002133	Cephalosporin;	Inactivation	DHA beta-lactamase
**MH618488.1_78**	48328	48465	+	900	22.3274	**tet(O/W/32/O)**	33.33	3007122	Tetracycline	Target protection	Tetracycline-resistant ribosomal
**MH618488.1_79**	48490	48750	+	450	24.2534	**OXA-573**	43.75	3005325	Carbapenem; cephalosporin;	Inactivation	OXA beta-lactamase
**MH618488.1_80**	48752	48958	+	2300	25.0238	**evgS**	44.44	3000833	Macrolide; fluoroquinolone	Antibiotic efflux	Major facilitator superfamily (MFS) efflux pump;
**MH618488.1_81**	48948	49250	+	2200	23.483	**ileS resistance to mupirocin**	50	3003730	Mupirocin-like antibiotic	Target alteration	Antibiotic-resistant isoleucyl-tRNA synthetase (ileS)
**MH618488.1_83**	50092	50757	+	400	26.1794	**catB8**	34.38	3002680	Phenicol antibiotic	Inactivation	Chloramphenicol acetyltransferase (CAT)
**MH618488.1_85**	51627	51845	+	450	24.2534	**rmtH**	46.15	3003198	Aminoglycoside antibiotic	Target alteration	16S rRNA methyltransferase (G1405)
**MH618488.1_86**	51847	52059	+	1700	29.6462	**LpeB**	43.48	3004100	Macrolide antibiotic	Antibiotic efflux	Resistance-nodulation-cell division (RND) antibiotic efflux pump

**MH618488.1_88**	54054	54488	-	850	26.1794	**hp1181**	28	3003964	Fluoroquinolone; tetracycline; nitroimidazole	Antibiotic efflux	Major facilitator superfamily (MFS) efflux pump
**MH618488.1_89**	54625	54879	-	1000	25.7942	**MCR-3.12**	37.04	3004693	Peptide antibiotic	Target alteration	MCR phosphoethanolamine transferase
**MH618488.1_91**	55161	55535	-	1900	26.9498	**MexK**	28.21	3003693	Macrolide; tetracycline	Antibiotic efflux	Resistance-nodulation-cell division (RND)
**MH618488.1_94**	56015	56254	-	500	23.8682	**APH (6)-Ia**	26.83	3002657	Aminoglycoside antibiotic	Inactivation	APH(6)
**MH618488.1_95**	57112	57624	-	725	25.409	**SRT-1**	25.3	3002493	Cephalosporin	Inactivation	SRT beta-lactamase
**MH618488.1_96**	57627	57809	-	200	23.8682	**tet(37)**	37.5	3002871	Tetracycline antibiotic	Inactivation	Tetracycline inactivation enzyme
